# Phase II study of radiotherapy combined with gemcitabine for locally advanced pancreatic cancer

**DOI:** 10.1038/sj.bjc.6602001

**Published:** 2004-06-29

**Authors:** T Okusaka, Y Ito, H Ueno, M Ikeda, Y Takezako, C Morizane, Y Kagami, H Ikeda

**Affiliations:** 1 Hepatobiliary and Pancreatic Oncology Division, National Cancer Center Hospital, 5-1-1 Tsukiji, Chuo-ku, Tokyo 104-0045, Japan; 2 Radiation Oncology Division, National Cancer Center Hospital, Tokyo, Japan

**Keywords:** pancreatic cancer, chemoradiotherapy, gemcitabine, radiosentitiser

## Abstract

Gemcitabine has been reported to be a potent radiosensitiser in human pancreatic cell lines. This study was conducted to evaluate the efficacy and toxicity of radiotherapy combined with gemcitabine for locally advanced pancreatic cancer. In all, 42 patients with pancreatic cancer that was unresectable but confined to the pancreatic region were treated with external-beam radiation (50.4 Gy in 28 fractions over 5.5 weeks) and weekly gemcitabine (250 mg m^−2^, 30-min infusion). Maintenance gemcitabine (1000 mg m^−2^ weekly × 3 every 4 weeks) was initiated 1 month after the completion of the chemoradiotherapy and continued until disease progression or unacceptable toxicity. Of the 42 patients, 38 (90%) completed the scheduled course of chemoradiotherapy. The major toxicity was leucopenia and anorexia. There was one death attributed to duodenal bleeding and sepsis. The median survival time was 9.5 months and the 1-year survival rate was 28%. The median progression-free survival time was 4.4 months. In 35 patients with documented disease progression at the time of analysis, 34 (97%) showed distant metastasis as the cause of the initial disease progression. The chemoradiotherapy used in this study has a moderate activity against locally advanced pancreatic cancer and an acceptable toxicity profile. Future investigations for treatment with more systemic effects are warranted.

Pancreatic cancer is the fourth leading cause of cancer death in the United States and the fifth leading cause in Japan. The statistics indicate a rapid increase in the number of deaths and the death rate due to pancreatic cancer in Japan, but the precise reasons are not clear, except for smoking. Pancreatic cancer in most patients is surgically unresectable at the time of diagnosis because of the difficulty of early detection of this disease. For patients with locally advanced pancreatic cancer, chemoradiotherapy has been accepted as standard treatment because the results of previous randomised trials have indicated that concurrent external-beam radiation therapy and 5-fluorouracil (5-FU) therapy results in a significantly longer survival time than radiotherapy (Moertel *et al*, 1969; Gastrointestinal Tumor Study Group, 1981) or chemotherapy alone (Gastrointestinal Tumor Study Group, 1988). In attempts to improve the efficacy of the treatment, numerous trials using modified approaches of chemoradiotherapy have been conducted (Chakravarthy and Abrams, 1997; Okada, 1999). However, there has not yet been a regimen that has demonstrated superiority over conventional chemoradiotherapy performed in randomised controlled trials.

Gemcitabine is a novel deoxycytidine analog, which has demonstrated significant clinical benefit and survival improvement compared with 5-FU in patients with advanced pancreatic cancer (Burris *et al*, 1997). Gemcitabine has also been shown to be a potent radiosensitiser in human pancreatic and other solid tumour cell lines (Lawrence *et al*, 1996; Shewach and Lawrence, 1996; van Putten *et al*, 2001), suggesting that the combination of radiotherapy and gemcitabine may improve survival in patients with locally advanced disease. A phase I trial that was conducted in our hospital determined the recommended dose of weekly gemcitabine for the phase II chemoradiotherapy trial to be 250 mg m^−2^ (Ikeda *et al*, 2002). We report our results of the phase II study that was conducted to clarify the efficacy and toxicity of concomitant chemoradiotherapy with gemcitabine in patients with locally advanced pancreatic cancer.

## PATIENTS AND METHODS

Patients eligible for this study had locally advanced pancreatic cancer for which they had not received any anticancer treatment. Each patient was required to meet the following eligibility criteria: pathological proof of adenocarcinoma of the pancreas; an Eastern Cooperative Oncology Group (ECOG) performance status of 0–2; adequate bone marrow reserve (white blood cell count ⩾4000 mm^3^, platelet count ⩾100 000 mm^3^, haemoglobin level ⩾10 g dl^−1^); adequate renal function (normal serum creatinine and blood urea nitrogen levels, and a creatinine clearance level ⩾60 mg min^−1^); a serum aspartate aminotransferase (AST) level <2.5 times upper normal limit (UNL); a serum alanine aminotransferase (ALT) level <2.5 times UNL; and written informed consent. Patients with obstructive jaundice were required to have a serum total bilirubin level of less than 2.0 mg dl^−1^ after biliary drainage. Pretreatment staging included ultrasonography and dynamic computed tomography (CT) scans of both the abdomen and the chest. The possibility for resection of the local tumour was assessed by dynamic CT and/or angiography. Obstruction or bilateral invasion of the portal vein and/or tumour encasement of the celiac or superior mesenteric arteries was considered to be unresectable. Patients were excluded if they met the following criteria: concomitant malignancy, pleural and/or peritoneal effusion, active ulcer of the gastrointestinal tract, active infection, severe heart disease, pregnant or lactating females, or other serious medical conditions. The goal was set at 40 eligible patients. This number of patients was planned using a design based on the assumptions that the median survival time in conventional chemoradiotherapy was 10 months, expected median survival time was 14 months, type I error was 5% (one-tailed) and statistical power was 70%.

Radiotherapy was delivered via a racetrack microtron (MM50, Scanditronix, Upsala, Sweden) with a 25 MV X-rays. A total dose of 50.4 Gy was delivered in 28 fractions over 5.5 weeks. All patients had treatment planning, CT scans (X-vision, Toshiba, Tokyo) and FOCUS (version 3.2.1, CMS, St Louis, MO, USA) was used as a radiotherapy treatment planning system. Clinical target volume (CTV) included the primary tumour, nodal involvement detected by CT scan and regional draining and paraaortic lymph nodes, which included the peripancreatic nodes, celiac and superior mesenteric axes. Planning target volume was defined as CTV plus a 10-mm margin. Four field techniques (anterior, posterior and opposed lateral fields) were used. Spinal cord dose was maintained below 45 Gy and ⩾50% of liver was limited to ⩽30 Gy, ⩾50% of both kidneys were limited to ⩽20 Gy.

Gemcitabine at a dose of 250 mg m^−2^ was given intravenously over 30 min starting 2 h before radiotherapy weekly for 6 weeks. This schedule was based on an *in vitro* study which revealed that gemcitabine induced its radiosensitising effect in cells within 2 h (Lawrence *et al*, 1997). Toxicity was assessed according to the National Cancer Institute – Common Toxicity Criteria version 2.0. When grade 3 haematological toxicity, serum creatinine of 1.5–2.0 times UNL, total bilirubin level of 3.0–5.0 times UNL, serum AST/APT of 5.0–10 times UNL and/or grade 2 nonhaematological toxicity (excluding nausea, vomiting, anorexia, fatigue, constipation, alopecia and dehydration) were observed, gemcitabine administration was omitted and postponed to the next scheduled treatment day. The radiotherapy was also suspended, and then resumed when the toxicities recovered. In patients who experienced the above adverse effects, dose reduction of gemcitabine to 200 mg m^−2^ was allowed in subsequent administrations. The combined treatment was discontinued when grade 3 leucopenia and/or neutropenia with high fever, grade 4 haematological toxicities after dose reduction of gemcitabine, serum creatinine of >2.0 times UNL, total bilirubin level of >5.0 times UNL, serum AST/APT of >10 times UNL, grade 3 or 4 nonhaematological toxicities (excluding nausea, vomiting, anorexia, fatigue, constipation, alopecia and dehydration), grade 4 vomiting, a total of 2 weeks of delay due to toxicity for any reason or tumour progression were observed. At 1 month after the completion of chemoradiotherapy, maintenance chemotherapy of gemcitabine at a dose of 1000 mg m^−2^ was administered as a 30-min intravenous infusion weekly for 3 weeks with 1-week rest until disease progression or unacceptable toxicity. Follow-up CT was performed within 1 week after the completion of chemoradiotherapy, and thereafter every 2 months to evaluate tumour response according to the WHO criteria (World Health Organization, 1979).

Progression-free and overall survival times were calculated from the first day of treatment using the Kaplan–Meier method (Kaplan and Meier, 1958). Serum CA 19-9 levels were measured monthly by a radioimmunometric assay using the Centocor radioimmunoassay kit (Centocor, Inc., Malvern, PA, USA).

## RESULTS

### Patients and treatments

In all, 42 patients were enrolled in the study between July 2001 and July 2002. Patient characteristics are listed in Table 1

**Table 1:** Patient characteristics

Number of patients	42
*Gender*	
Male	19 (45%)
Female	23 (55%)
	
*Age (years)*	
Median (range)	59 (43–73)
	
*ECOG performance status*	
0	12 (29%)
1	30 (71%)
	
*Tumour location*	
Head	21 (50%)
Body–tail	21 (50%)
	
*CEA (ng ml*^−*1*^)	
Median (range)	11 (1.0–62.7)
	
*CA19-9 (U ml*^−*1*^)	
Median (range)	2775 (1–15 620)

ECOG=Eastern Cooperative Oncology Group; CEA=carcinoembryonic antigen; CA19-9=carbohydrate antigen 19-9.

. A total of 38 patients (90%) received the full regimen of chemoradiotherapy, and the remaining four patients (10%) discontinued the treatment after 18.0–45.0 Gy. The reasons for the treatment discontinuation were elevated serum ALT of >10 times UNL (two patients), duodenal bleeding (one), and patient's refusal of treatment due to general fatigue (one). After discontinuation of the chemoradiotherapy, the two patients who showed the ALT elevation suspected as gemcitabine-related toxicity received chemoradiotherapy using 5-FU, and the other two patients underwent only supportive care. Of 241, 30 (12%) planned gemcitabine injections (0.7 injections per patient) were omitted owing to adverse events including grade 3 or more leucopenia and/or neutropenia, grade 2 fever, grade 2 skin rash and patient's refusal due to nausea, vomiting or fatigue. In three patients who showed grade 4 leucopenia and/or neutropenia, the dose of gemcitabine was modified in subsequent injections. Maintenance chemotherapy was initiated in 23 of the 38 patients who completed the full regimen of chemoradiotherapy. Of the remaining 15 patients, seven showed deterioration of general condition due to disease progression before initiating the chemotherapy, seven refused the treatment due to appetite loss (4) or general fatigue (3) and one transferred to another hospital (1).

### Response and survival

Tumour response was determined in 40 patients. Two patients were excluded from the protocol efficacy analysis because their treatment was switched over to chemoradiotherapy using 5-FU before the response evaluation due to the ALT elevation. Nine patients (21%) achieved a partial response, 26 (62%) remained stable and five (12%) showed progressive disease demonstrated by the development of distant metastases. No patients could undergo tumour resection even after the completion of chemoradiotherapy because of infiltration of the adjacent large vessels. In 22 (76%) of the 29 patients with a pretreatment serum CA19-9 (carbohydrate antigen 19-9) level of 100 U ml^−1^ or greater, the level was reduced more than 50% within 14 weeks after initiation of treatment.

A total of 35 patients documented disease progression at the time of analysis. The initial sites of disease progression are listed in Table 2

**Table 2:** Patterns of initial disease progression

**Local**	**No. (%)**
Distant metastasis	33 (94)
Peritoneum	17 (49)
Liver	15 (43)
Lymph node	1 (3)
Ovary	1 (3)
Bone	1 (3)
Local and distant metastasis	1 (3)

**Figure 1:**
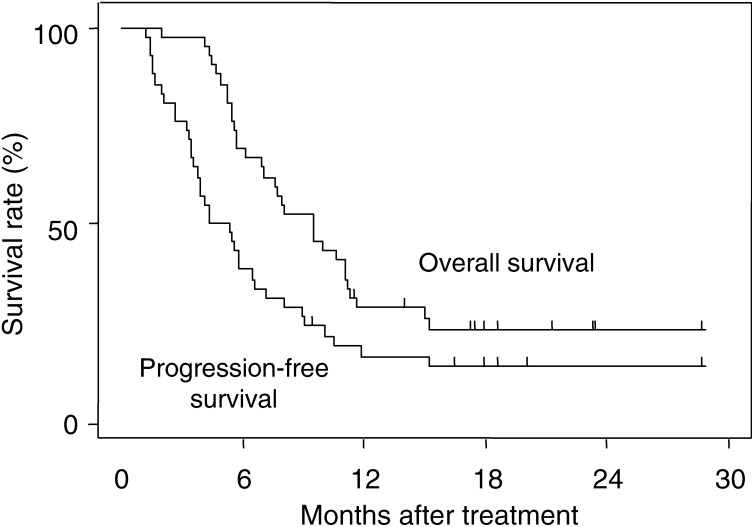
Progression-free survival and overall survival curves of patients with locally advanced pancreatic cancer receiving radiotherapy with gemcitabine.

. The pattern of failure was distant metastases in 33 patients (94%), local–regional recurrence in one patient (3%) and both in one patient (3%). The median progression-free interval and the median survival time were 4.4 and 9.5 months, respectively. The overall 1- and 2-year survival rates were 28 and 23%, respectively (Figure 1

).

### Toxicity

The acute toxicity is summarised in Table 3

**Table 3:** Acute toxicity

**Grade**	**1 (%)**	**2 (%)**	**3 (%)**	**4 (%)**
*Haematological toxicity*				
Leucocytopenia	3 (7)	17 (40)	21 (50)	1 (2)
Neutropenia	9 (21)	15 (36)	11 (26)	3 (7)
Thrombcoytopenia	22 (52)	2 (5)	1 (2)	0 (0)
Anaemia	21 (50)	17 (40)	0 (0)	1^a^ (2)
				
*Nonhaematological toxicity*				
Total bilirubin	10 (24)	5 (12)	1 (2)	0 (0)
AST	14 (33)	5 (12)	1 (2)	0 (0)
ALT	15 (36)	11 (26)	4 (10)	0 (0)
ALP	15 (36)	5 (12)	0 (0)	0 (0)
Creatinine	0 (0)	0 (0)	0 (0)	0 (0)
Anorexia	9 (21)	5 (12)	10 (24)	14 (33)
Nausea	11 (26)	11 (26)	9 (21)	0 (0)
Vomiting	10 (24)	7 (17)	0 (0)	0 (0)
Diarrhoea	1 (2)	1 (2)	0 (0)	0 (0)
Mucositis	0 (0)	0 (0)	0 (0)	0 (0)
Duodenal ulcer	0 (0)	0 (0)	0 (0)	1^a^ (2)
Fatigue	17 (40)	14 (33)	2 (5)	0 (0)
Skin rash	0 (0)	1 (2)	0 (0)	0 (0)
Infection	0 (0)	0 (0)	0 (0)	1^a^ (2)

AST=aspartate aminotransferase; ALT=alanine aminotransferase; ALP=alkaline phosphatase.

a One patient died of duodenal bleeding and sepsis.

. The haematological toxicity was relatively brief and reversible in most patients. Grade 3–4 leucopenia and neutropenia occurred in 22 (52%) and 14 (33%) of the patients, respectively. Grade 3 thrombocytopenia occurred in one patient (2%) on the day after the chemoradiotherapy completion. The patient, who showed grade 4 anaemia, suffered catastrophic duodenal bleeding requiring embolisation under angiography. She exhibited cholangitis and sepsis subsequently and died on day 63.

The most common nonhaematological toxicity was anorexia, which was observed in 38 patients (90%). In total, 14 patients (33%) required intravenous hyperalimentation. In all, 33 patients (79%) complained of fatigue and one of them refused continuation of the chemoradiotherapy. Nine patients (21%) experienced grade 3 nausea. Liver function abnormality was another major adverse effect. Four patients (10%) showed grade 3 elevation of serum transaminase levels. Two of them discontinued the treatments after 19.8 and 21.6 Gy, respectively, due to serum ALT elevation of 10 times UNL according to the protocol criteria (maximum level: 452 and 435 IU l^−1^), although the serum ALT levels of both recovered to the grade 1 levels 4 days after discontinuation of the treatment. We suspected that the ALT elevation in these two patients was gemcitabine-related toxicity because it was never reproduced after their treatment was switched over to chemoradiotherapy using 5-FU. One patient suffered unexpected acute abdominal pain requiring morphine 2 months after the completion of the chemoradiotherapy and was diagnosed with perforation of pancreatic pseudocyst into the duodenum. This pain disappeared completely by only medical management within 1 week. No patients experienced any symptoms considered to be late toxicity as of the time of analysis.

## DISCUSSION

Based on previous randomised trials (Moertel *et al*, 1969; Gastrointestinal Tumor Study Group, 1981; Gastrointestinal Tumor Study Group, 1988), concurrent external-beam radiotherapy and 5-FU have been generally accepted as the standard treatment for locally advanced carcinomas. To intensify the treatment efficacy, various anticancer agents and radiation schedules are being investigated in clinical trials of chemoradiotherapy (Roldan *et al*, 1988; Seydel *et al*, 1990; Wagener *et al*, 1996; Thomas *et al*, 1997; Prott *et al*, 1997; Okusaka *et al*, 2001). However, marked improvement in their survival has not been observed. In an attempt to optimise radiosensitisation, radiotherapy with protracted 5-FU infusion has been examined recently, but the median survival times were similar to those observed in previous studies (Ishii *et al*, 1997).

Gemcitabine has been expected to be an agent that improves the outcome of chemoradiotherapy for locally advanced pancreatic cancer because it is a chemotherapeutic drug having meaningful palliative and prognostic impact against advanced pancreatic cancer, and it is also a potent radiosensitiser. Several experimental studies have shown that more than one mechanism leads to the potentiation of radiation-induced cell killing by gemcitabine (Lawrence *et al*, 1996; Shewach and Lawrence, 1996; van Putten *et al*, 2001). In clinics, various phase I studies for radiotherapy with gemcitabine have been conducted (McGinn *et al*, 2001; Pipas *et al*, 2001; Wolff *et al*, 2001; Ikeda *et al*, 2002; Poggi *et al*, 2002), although the efficacy and safety of this combination have not been fully elucidated in phase II trials. A phase I trial that was conducted in our hospital determined the recommended dose of weekly gemcitabine in the phase II chemoradiotherapy trial to be 250 mg m^−2^, because three of the six patients give a dose of 350 mg m^−2^ of gemcitabine demonstrated dose-limiting toxicities involving neutropenia/leucopenia and elevated transaminase (Ikeda *et al*, 2002).

The toxicity associated with radiotherapy with gemcitabine was relatively severe in this phase II study. Grade 3–4 leucopenia and neutropenia were observed in 52 and 33% of the patients, respectively, although none of the patients showed neutropenic fever. Nausea and anorexia were the most serious non-haematological toxicities in this treatment; 73% of the patients experienced various degrees of nausea and 33% required intravenous hyperalimentation. In all, 78% of the patients complained of general fatigue and one patient (2%) refused continuation of the treatment because of this adverse effect. These troublesome toxicities observed in this study seem to be more frequent and more severe compared with those in 5-FU-based chemoradiotherapy (Ishii *et al*, 1997). There was one death attributed to duodenal bleeding, which was arrested by transcatheter arterial embolisation, but deterioration of the general condition and lethal sepsis were induced subsequently.

The present study, in which 42 patients with locally advanced pancreatic cancer were treated with radiotherapy and weekly gemcitabine, documented a marginal impact on patient survival; the median survival time of 9.5 months is comparable to that in patients receiving conventional chemotherapy using 5-FU. However, the incidence rate of distant metastasis at the time of disease progression was remarkably higher with this treatment (97%) as compared to that with 5-FU-based chemoradiotherapy, which was reported to be 50% in our previous study (Ishii *et al*, 1997). This suggests that gemcitabine at a dose of 250 mg m^−2^ is a potent radiosensitiser for controlling local disease, but its ability as a chemotherapeutic agent is insufficient to counteract systemic tumour spread. To improve prognosis for these patients, future investigations for treatment with more systemic effects are warranted.

In an effort to increase capacity for systemic therapy, reduction of the radiation field has been attempted. Investigators at the University of Michigan elected to radiate the primary tumour alone, without the inclusion of regional lymph nodes, and administer full-dose gemcitabine concurrently, because the use of full-dose gemcitabine requires reduction of the radiation dose, based on their prior clinical experience (McGinn *et al*, 2001; Muler *et al*, 2004). Reduction of the radiation field may be one of the strategies not only for intense systemic therapy but also for decreasing the troublesome gastrointestinal toxicity often observed in our study; our recent retrospective study showed that a larger planning target volume for irradiation was only a significant predictor of severe acute intestinal toxicity in patients treated with chemoradiotherapy using gemcitabine (Ito *et al*, 2003).

Crane *et al* (2002) retrospectively compared the toxicity and efficacy of concurrent gemcitabine-based chemoradiation with those of concurrent 5-FU-based chemoradiation in patients with unresectable pancreatic cancer treated in the University of Texas MD Anderson Cancer Center. In the study, there was a significantly higher severe toxicity rate in patients treated with gemcitabine than in those with 5-FU, although the median survival times were similar between the two arms (gemcitabine *vs* 5-FU: 11 *vs* 9 months). They concluded that concurrent gemcitabine and radiotherapy could be an extremely difficult combination to administer safely, with a very narrow therapeutic index. Recently, investigators in Taiwan reported favourable results for radiotherapy with concurrent gemcitabine administration (600 mg m^2^ week^−1^ for 6 weeks) in a small randomised study (Li *et al*, 2003). The gemcitabine-based chemoradiotherapy showed a significantly better median survival time (14.5 months) and a comparable toxicity profile in comparison with the 5-FU-based chemoradiotherapy (7.1 months). However, the number of enrolled patients in this study was only 16–18 in each arm. The results need further confirmation by larger multi-institutional clinical trials.

In summary, the chemoradiotherapy used in this study has a moderate activity against locally advanced pancreatic cancer and an acceptable toxicity profile, but appears to have more frequent acute toxicities compared with conventional chemoradiotherapy using 5-FU. Most patients who underwent this therapy demonstrated rapid appearance of distant metastasis. To explore innovative approaches for locally advanced pancreatic cancer, future investigations for treatment with more systemic effects and less toxicity are needed.
